# Prescribing Vitamin-K-Antagonists Versus Direct Oral Anticoagulants Among Bavarian General Practitioners: A Qualitative Study

**DOI:** 10.1177/11786329251341083

**Published:** 2025-06-21

**Authors:** Nikoletta Zeschick, Julia Gollnick, Julia Muth, Franziska Hörbrand, Peter Killian, Norbert Donner-Banzhoff, Thomas Kühlein, Maria Sebastião

**Affiliations:** 1 Institute of General Practice, Friedrich-Alexander-University Erlangen-Nürnberg, University Hospital Erlangen, Germany; 2 Institute of General Practice/Family Medicine, Philipps University of Marburg, Germany; 3 Association of Statutory Health Insurance Physicians, Bavaria, München, Germany

**Keywords:** Wirkstoffvereinbarung, active substance agreement, DOACs, VKAs, drug costs

## Abstract

**Background:**

Direct oral anticoagulants (DOACs) have been increasingly prescribed instead of vitamin-K-antagonists (VKA) although VKAs cost considerably less than DOACs. In 2014, a new system for drug expenditures, the Wirkstoffvereinbarung (WSV, Active substance agreement), was implemented in Bavaria, Germany to control pharmaceutical expenditures transparently. Achieving the targets for the VKAs set by the WSV was difficult for general practitioners (GPs). We explored the determinants of prescribing VKAs (specifically phenprocoumon) versus DOACs.

**Methods:**

Qualitative interviews (n = 18) and two small group discussions (n = 10) were conducted with GPs. For the qualitative content analysis, we formed a system of categories based on the domains of the Theoretical Domains Framework (TDF).

**Results:**

Participants actively weighed various factors when deciding between prescribing phenprocoumon or DOACs. Costs played a subordinate role although all participants were aware that DOACs come at a higher cost than phenprocoumon. Trend reports served as a tool for GPs to assess their prescribing practices, however did not lead to a change in prescribing behaviour. The interviewees had a very heterogeneous view of safety, effect, and evidence of phenprocoumon or DOACs. The cooperation of the patients is crucial. Time is a significant challenge for participants when initiating patients on or switching them to phenprocoumon, which is especially problematic as all of the patients discharged from the hospital are put on DOACs.

**Conclusions:**

GPs are caught between economic requirements, patients’ wishes, and good collegial cooperation when deciding for or against phenprocoumon. As oral anticoagulant therapy (OAC) is mostly initiated in the hospital, and as physicians almost exclusively prescribe DOACs there, participating GPs feel overchallenged in reaching the targets set by the WSV.

**Trial registration number:**

Main ID: DRKS00019820 (German Register of Clinical Studies and World Health Organization).

## Introduction

Atrial fibrillation (AF) is the most common arrhythmia in clinical practice^1^ and is associated with an increase of ischemic stroke.^1,2^ Oral anticoagulation (OAC), including vitamin K-antagonists (VKAs) and direct oral anticoagulants (DOACs), significantly reduces the risk of stroke in patients with AF.^3^ In Germany, the almost exclusively used VKA is phenprocoumon and was long considered the standard treatment. Over the past years, direct oral anticoagulants (DOACs) were on the rise.^4^ From 2011 to 2016, four DOACs were approved for this indication in Germany: dabigatran, rivaroxaban, apixaban, and edoxaban. This approval was based on Randomized Controlled Trials (RCTs) comparing DOACs to warfarin (VKA), where the benefits of DOACs over warfarin could be demonstrated. It is usually assumed that study evidence obtained from warfarin also applies to the VKA phenprocoumon, almost exclusively used in Germany. To our knowledge, there is no RCT that compared DOACs to the VKA phenprocoumon. However, several so-called real-world studies with phenprocoumon exist that do not reveal any relevant advantages in terms of benefits and harms, and in some cases even have disadvantages compared to DOACs.^5,6^ A clear advantage of DOACs is that they are more comfortable for physicians and patients alike as testing for the degree of anticoagulation is neither necessary nor feasible. On the other hand, a clear disadvantage between DOACS and VKAs lies in the average drug costs: these were in 2016 about 20 times higher for DOACS than for VKAs. Nevertheless, DOACS are increasingly being described in Germany since then.^7^

**Figure 1.**
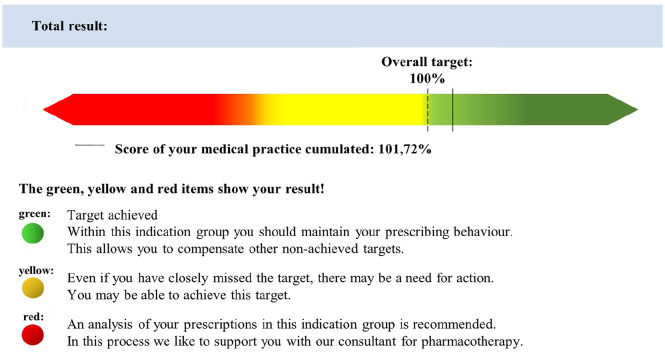
Excerpt from the WSV: trend reports of the ASHIP, Bavaria displaying the total target achievement (cumulated).^10^

To ensure the economic efficiency of healthcare in the ambulatory sector, various surveillance instruments have been developed. In 2014, the sick funds, and the regional Associations of Statutory Health Insurance Physicians (ASHIPs) of Bavaria, one of the countries in the federal Republic of Germany, newly installed the “Wirkstoffvereinbarung” (WSV; *English*: Active Substance Agreement).^8^ The WSV replaced the “Richtgrößenprüfung” (RGP; *English*: Prescribing Target Scheme).^9^ The WSV is based on the principle of directing prescribing behaviour toward the requirements of an economic efficiency principle. The WSV was revised in 2017 (WSV2.0) and 2020 (WSV 3.0). In WSV 2.0 for 31 indication areas 32 drug targets were defined. The targets either refer to a percentage of recommended drugs or a predefined percentage of generic prescriptions. The measurement category is the Defined Daily Dose (DDD). For example, GPs had to prescribe 53.0% DDDs of VKAs in WSV 2.0 as recommended drugs among oral anticoagulants. In the last quarter of 2019, only a rate of 27.57% was achieved by GPs. Each quarter, physicians receive a detailed trend report visualized by a traffic light system (green/yellow/red) as feedback on their prescribing behaviour (Figure 1). This way, the physicians see where they can prescribe more cost-efficiently.

The *WirtMed study*, funded by the Innovation Fund of the Federal Joint Committee (*Gemeinsamer Bundesausschuss*, grant number: 01VSF17016), deals with the (further) development of different systems to direct and control cost-efficient drug prescriptions in the light of the legal requirements of the German Social Code (Sozialgesetzbuch V; SGB V §12).^11^ In our analysis we found that none of the participating GPs achieved the targets for the VKAs, some physicians being yellow, some in the red range.^12^ This paper focuses on the analysis of the data to explore the determinants of prescribing VKAs (specifically phenprocoumon) versus DOACs.

## Design

### Study Design

A qualitative study design was chosen to comprehensively reflect the physicians’ perspective. From 10/2019 to 03/2020 semi-structured single interviews and focus groups were conducted. The single interviews allowed more depth, while the focus groups aimed to identify new aspects through joint discussion. The ethics committee of the Friedrich-Alexander-University Erlangen-Nürnberg approved the study (File number 65_19 B).

### Recruitment and Setting

The Bavarian ASHIP approached 697 GPs physicians with an overall target achievement of ⩾90% according to their trend reports in the quarters of 2017. The overall target achievement does not refer exclusively to the VKA-DOAC target but to all targets combined, which include various medication targets set by the agreement. Physicians who reached a lower overall target achievement were excluded in our study and included in another sub-project.^13,14^ There were no other exclusion criteria. For the qualitative evaluation of the WSV, we planned on n = 20 interviews and n = 5 group discussions consisting of five to eight physicians. We decided to contact a large number of physicians because we anticipated that only a small proportion would respond. This approach ensured that we could still reach enough participants for the study. All physicians who expressed interest in the study were invited to participate, resulting in 56 physicians. However, for the purposes of this analysis, we focused exclusively on general practitioners (GPs), as they are the primary group dealing with the challenges related to the DOAC-VKA target.

After the initial response, further contact was exclusively via the Institute of General Practice at the Uniklinikum Erlangen (IGP UK-ER). Interviewers and physicians did not know each other beforehand.

### Data Collection

Researchers from the IGP UK-ER and the Universität Marburg (pedagogue/psychologist, and pharmacist) developed the interview-, and (small) group discussion guides (Additional file 1 in the Supplemental Material). They comprised four topic sections: (1) general information on the person, and practical experience as a physician (eg, duration of outpatient work), (2) general information on prescribing behaviour (eg, dealing with new medicines, and/or medicines initiated by others), (3) overall situation regarding WSV (eg, advantages, and disadvantages), and (4) conclusion (eg, other aspects, an overall assessment). DOACS/VKAs were addressed in the various sections throughout the interviews. The guides were pre-tested with four physicians. All participants gave written informed consent.

The in-depth interviews (n = 18) were conducted by two female researchers in each physician’s practice. NZ was the main interviewer at every interview. NDB, TK, and MS have extensive knowledge in qualitative research. Before data collection, NZ was trained in qualitative data collection. The two (small) group discussions followed the same procedure, except for omitting some sub-questions. The (small) group discussion took place at the IGP UK-ER (SGD1, participants n = 8) and in a rented room (SGD2, participants n = 2). The interviews (average 60 minutes) and (small) group discussions (about 120 minutes) were digitally audio-recorded and later transcribed verbatim. The transcription was done by an external provider. Afterward, NZ checked all transcripts twice and corrected any errors. In addition, protocols of the sessions were written to include, for example, disturbances or first ideas. Personal information about participants were anonymized following a pre-defined protocol.^15^ Transcripts were not provided to participants.

### Data Analysis

All categories were continuously discussed by the project team, meeting the standard of consensual validation. During the study, a research diary was kept in which NZ noted her own role and possible influence during data collection, but also initial ideas for the analysis. The content of the research diary was regularly discussed with MS. The idea to further analyze the prescription of VKAs and DOACs came from these initial ideas. The analysis was carried out by NZ and MS, using the software program MAXQDA Plus 2020.^16^ The first phase of reading and re-reading each transcript as well as listening to interview recordings to become familiar with the content was part of the first analysis.^12,17^ During this first analysis, the DOACs became apparent as a problematic substance group, and passages throughout the material were marked for further analysis by NZ. In the secondary analysis, we used these pre-selected text sections. MS formed a system of categories based on the domains of the Theoretical Domains Framework (TDF). The TDF is a validated framework to understand facilitators and barriers to changing behaviour.^18,19^ The framework consists of 14 domains that split into 84 determinants.^18^ The behaviour of our interest was the prescribing of VKAs versus DOACs (including initiation and renewal of prescription). NZ and MS applied the categories to the pre-selected text sections and afterward to the whole material to make sure not to miss anything. In the last phase we interpreted the data by finding associations between themes. The reporting of the study is following the “Consolidated criteria for reporting qualitative research” (COREQ).^20^

## Results

### Study Population

Twenty-eight GPs (19 men, 9 women) of various age groups participated in the study (Table 1). Individual and group practices, as well as different practice locations (rural, small, medium, and large cities), were represented.

**Table 1.** Sample characteristics.

Characteristics	Total sample (N = 28)		Men (n = 19)		Women (n = 9)	
	n	%	n	%	n	%
*Age*						
41-50 y	11	37	7	37	4	45
51-60 y	7	26	5	26	2	22
60+ y	10	37	7	37	3	33
*Years of total working experience*						
1-14	1	4	0	0	1	11
15-29	21	75	15	79	6	67
30-45	6	21	4	21	2	22
*Years of working experience in ambulatory care*						
1-14	12	43	8	42	4	44
15-29	12	43	8	42	5	56
30-45	4	14	3	16	1	11
*Type of practice*						
Single practice	14	50	7	37	7	78
Community practice	11	39	9	47	2	20
Other^1^	3	11	3	16	1	10
*Location of practice*						
Countryside^2^	8	29	5	26	3	33
Small/medium-sized town^3^	11	39	9	47	2	22
Big city^4^	9	32	5	26	4	45
*Type of employment*						
Full-time	25	89	17	90	8	89
Part-time	3	11	2	10	1	11

1 Other = group practice, single practice with employed physicians.

2 Number of inhabitants below 5000.

3 Number of inhabitants up to 100 000.

4 Number of inhabitants above 100 000.

Almost all TDF domains were seen to be relevant. The ones that are presented below were emphasized by the participants, for example, through the frequency of occurrence of each domain across all the transcripts.

### Knowledge and Belief About Consequences

Since the two TDF domains knowledge about therapy safety and effects go hand in hand with the consequences, the domains will be presented together. The participants’ opinion about the evidence differed, some physicians stated that the studies with DOACs are not conclusive enough or show little to no advantage (I18) others stated that DOACs have the “*higher evidence*” level (I13) and are the therapy of choice according to guidelines (I13).

Participating GPs varied in their individual assessments and experiences with VKAs or DOACs, which influenced their prescribing behaviour. The primary concern revolved around the differing evaluations of the risk of bleeding. Some interviewees expressed concerns about potential safety issues with VKAs, noting instances of bleeding complications despite precautions (I13). Whereas others reported side effects such as “*unexpected bleeding*” while patients were on DOACs (I17). Consequently, they reported to have changed the medication of these patients back to VKAs, which resulted in no further problems (I17; I18). Participating GPs further highlighted the advantage that DOACs bring faster results with initial dosing compared to VKAs: “*patients with fresh leg vein thrombosis, I don’t want to start with Marcumar [phenprocoumon] when I know that with Xarelto [DOAC] I can have the effect in two hours, right?*” (I11). However, the long-term effects of DOACs were evaluated as uncertain: “*With the DOACs, nobody knows what the results will be in ten years*” (I20) whereas the effects of VKAs are well known, even an extended period of time (I20). Personal biases or past clinical experiences influence prescribing decisions by shaping intuitive judgments (I03), risk perceptions (I13), and confidence (I01) in certain treatments. These factors can lead to preferences for medications, avoidance of specific drugs due to negative past experiences, or reliance on anecdotal evidence rather than guideline-based recommendations. In the TDF-domain “knowledge,” uncertainties in dosage were not evident for either VKAs or DOACs, leading to the conclusion that the prescribing behaviour in our sample was not influenced by a lack of knowledge in this regard.

**Key message:** GPs’ prescribing behaviour was influenced by differing opinions on the evidence for DOACs, personal experiences with bleeding risks, and the need for faster onset in acute cases. While DOACs were valued for their immediate effect, concerns about their long-term safety contrasted with the well-established effects of VKAs.

### Environmental Context and Resources

Time and financial considerations are significant challenges for participants. Initiating patients on VKA is “*time consuming*” (I03). Eventually, GPs may opt to advise *“just take your DOAC*” (I03). Switching from DOAC to VKA is stated to be impossible due to the substantial effort involved (I02) for the Participants. Those GPs who attempted to make the switch post-hospitalization confirmed that it is a “*huge discussion*” with patients (I06).

All participating GPs were aware that DOACs come at a higher cost than VKAs. Nevertheless, they emphasized that this calculation does not factor in their own work (I06 ). Contacting patients, conducting blood tests, and needing staff to observe the patients – all of these consume time and resources which are not explicitly compensated for. This aspect was particularly stressed when INR-testing of patients necessitates home visits (I14, I03). However, this was also assumed to be a relevant point for patients: “*DOACs undeniably offer a much more comfortable therapy experience for the patient*” (I10).

Time was also related to communication (TDF domain: Skill). However, it was noticeable that the participating GPs did not attribute the issue to a communication problem per se but rather to insufficient time for the necessary explanations to patients.

They also noted that patients who are dissatisfied with their care go to seek other physicians, as patients have a free choice of physicians in the German healthcare system. In this context, dissatisfaction was believed to often arise from being prescribed VKAs (I20). The participants perceived the adverse economic implications and the resulting potential sanctions as less meaningful compared to the possible loss of patients.

**Key message:** Although all participating GPs were aware of the higher costs of DOACs, they mostly believed than when factoring in other factors like time and repeat examinations, VKAs are more expensive for the healthcare system. Loosing patient was more threatening than potential sanctions.

### Memory Attention and Decision Process

Participating GPs actively weighed various factors when deciding between prescribing VKAs or DOACs. Foremost among these considerations, they mentioned the cooperation of the patients. When it comes to prescribing VKAs, it’s emphasized that “*you need a highly compliant patient*” (I03). Lifestyle and occupation are an important argument against VKAs. For instance, patients with lots of work-related travel duties, do not make it to regular controls of INR: “*So I am compelled to prescribe a DOAC, whether it makes sense or not.*” (I09). They also addressed that poor vein conditions may be the reason for choosing DOACs (I12; I18). The patient’s preferences or characteristics take precedence over cost-effectiveness. When choosing the actual drug, on the other hand, the interviewees take the costs for the different types of drugs into account and opt for the cheaper alternative (I15). Meaning, once GPs decide to prescribe a DOAC, the selection of the specific DOAC (dabigatran, rivaroxaban, apixaban, or edoxaban) is influenced by cost.

There are also instances where VKAs remain the only viable option: “*DOACs are not approved for every scenario*” (I09; I19), such as mechanical heart valves. So, there are still cases where a VKA is the only option (I12).

**Key message:** GPs prioritize patient cooperation, lifestyle, and medical conditions when choosing between VKAs and DOACs, while cost considerations mainly influence the specific DOAC prescribed.

### The Benefits of VKA Monitoring (TDF: Beliefs About Capabilities)

VKA enables physicians to monitor the intake of the medication: “*When I observe that it’s not yielding results, I can inquire why? Is the patient not taking the tablets or are they taking it incorrectly? Because I can measure it*” (I11). This quote also shows that phenprocoumon may offer a higher level of safety in treatment. Most participating GPs affirmed this as the primary advantage (I20, I14). Not only the physicians can monitor the medication status, but also the patients, reflecting a significant enhancement in patient engagement and empowerment. Some physicians even characterized their patients as enthusiastic advocates of phenprocoumon, who diligently maintain their own records (I20). The routine blood checks also ensure continuous doctor-patient interaction, even beyond the actual illness (I11). When a patient is “*doing well*” (I20) with VKA, participating GPs continue to prescribe it (I09, I14).

### Social/Professional Role and Identity

The interaction with other prescribers became evident as a source of tension. The participants reported that all the patients discharged from the hospital are put on DOACs (I20). This applies not only to those newly initiated on DOACs, but also to patients who were previously on VKA: “*The problem arises when people return from the hospital [. . .] they have been switched from Marcumar [phenprocoumon] to a DOAC*.” (I14, pos. 111). Hospitals were seen as the initiators of DOACs over VKA and therefore drive the use of expensive prescriptions, which GPs have to implement (I03). Reverting those patients (back) to VKA is seen as “*practically unfeasible*” (I20). The participating GPs also described that they are torn between professional societies: *The German Society of General Practice and Family Medicine says: ‘No, do it with Marcumar.’ The cardiologists say: ‘But the risk of cerebral haemorrhage is much lower [with DOACs]!”* (I3).

The participating GPs’ personal convictions played an important role in their prescribing practices. Two distinct groups emerged among them, irrespective of the economic target. The main reasons for these two sides appeared to be the differing assessments of the evidence and possible side effects. The group that prefers VKAs, for instance, justifies this by pointing to deficiencies in the sample: “*Personally, I’m not entirely convinced [by DOACs] based on the patients I encounter. They would never have been included in a DOAC study*.” (I11). Despite their reservations, they occasionally prescribed DOACs, depending on patients’ characteristics. The other group was opposed to VKAs: “*I can no longer endorse Marcumar [phenprocoumon] with a clean conscience.*” (I10). Particularly within this group, the WSV was viewed as a restriction on therapeutic autonomy and the VKA-target was deemed as outdated (I20).

**Key message:** GPs face tensions with hospitals, which predominantly prescribe DOACs, making it difficult to revert patients to VKAs. Additionally, conflicting guidelines and personal convictions lead to divided prescribing preferences, with some GPs favouring VKAs due to safety concerns and others rejecting them entirely.

### Social Influences

The availability of information on drugs in mainstream media (I10) or within the social environment can influence patients and their preferences: “*You can read about it everywhere. Whether it’s true or not. They [=patients] talk to others, let’s say, who are the same age, who perhaps also have this medication. If someone comes to you [=GP] and you say: ‘Yes, you should take Marcumar [phenprocoumon]’, they will look at you disapprovingly. [. . .] So you don’t really have any options left.*” (I20).

### Reinforcement, Emotion and Intentions

Emotional responses to previous patient outcomes may unconsciously affect decision-making (I13), contributing to variability in prescribing practices. Trend reports served as a tool for GPs to assess their own prescribing behaviour. However, even when the reports indicated that they were in the yellow or red range for DOACs, participating GPs displayed little to no intention to increase their prescription of VKAs in the future. A sense of resignation emerged, as they felt powerless to effect change: “*Sometimes it’s frustrating when you can’t change anything. That’s just the way it is.*” (I02) or “*I feel trapped. Too many patients are now on DOACs. And changing their medication is simply not feasible for patients.*” (I18). This sentiment is tied to their perception that hospitals play a significant role in prescribing DOACs. If patients do not encounter issues with taking VKA or experience side effects, the interviewees will continue to prescribe it. Otherwise, they will only initiate treatment with VKA if the circumstances align appropriately. Although the participating GPs did not meet the target, they had little to no fear of sanctions. They asserted that their ethical compass guides their actions (I13) and viewed thorough documentation as a protective measure (I09).

## Discussion

Our study revealed the determinants that can influence GPs’ prescribing behaviour for VKAs (here specifically: phenprocoumon) compared to DOACs in the context of restrictions by a body surveying prescribing costs. The participants reported that almost all the patients with an indication for oral anticoagulation discharged from the hospital are put on DOACs instead of VKAs. When other colleagues prescribe DOACs, or when a VKA has already been administered over an extended period, the prescribing regimen is mostly maintained by them. They were aware of the higher costs associated with DOACs. Nevertheless, cost-efficient considerations as such have a marginal impact on their decision-making. Lack of time and the cooperation of the patients pose significant challenges for GPs, when initiating VKA. This implies that patients’ characteristics play a role in the decision-making process. The interviewed GPs held different opinions regarding the evidence on the differences between DOACs and VKAs and had varied experiences with perceived side-effects of both, ranging from none to numerous.

### Comparison to the Existing Literature

After the market launch of DOACs in Germany, there was a continuous increase in DOAC prescriptions and a simultaneous decrease for a VKA.^21^ This trend was seen worldwide.^22-24^ In the experience of our GPs, OAC treatment initiation in hospitals almost exclusively involves the use of DOACs, and at times, patients already on OAC are even switched from VKAs to DOACs during their hospital stay. In one of our former analysis, GPs reported that DOACs are heavily promoted in hospitals which creates significant pressure on GPs to align with hospital prescribing practices.^12^ The change of medication often occurs without communication or cooperation with GPs, despite them being responsible for the long-term prescriptions and their costs in outpatient care.^12^ This highlights the important role of hospital physicians in determining prescription choices.^12^ Communication between primary and secondary care was identified as a key challenge in other studies as well.^25^ In general practice, switching OAC therapy is rather uncommon in both directions, and when it does occur, patients typically are transitioned from VKAs to DOACs. According to a Dutch study, a switching rate was found to be 5% among prevalent users and 6% among new starters in general practice.^26^ Only a small proportion of patients who have switched to DOACs switch back to VKAs.^27^ While therapeutic convenience was one of the most important reasons for switching to DOACs,^27^ our study found it to be a more influential factor at initiation rather than when changing treatments. In a survey conducted among office-based physicians in Germany (36% of them GPs), it was discovered that physicians tend to prescribe VKAs more frequently and DOACs less frequently than their preferences would suggest.^28^

Multiple factors intertwine while making decisions on OAC initiation and anticoagulant switch.^29^ As well as in other studies, knowledge and experience with OAC therapy play an important role in decision making.^29^ Studies have shown that physicians have difficulty quantitatively assessing the benefits and harms of medication.^30^ The WSV is based on evidence and not exclusively on costs. It would therefore be necessary to support physicians with regard to the principles of evidence-based medicine and the subtleties of study results. To support GPs, at the end of each trend report, consulting and training opportunities through ASHIP are pointed out. The seminars are free of charge and had many participants. However, there was no evaluation to check what influence the information had on prescribing behaviour.

Lack of time was identified as a major barrier to prescribing VKAs more frequently or switching to them more often. This lack of time purportedly is affecting both GPs and patients, who must attend practices more frequently for INR monitoring. In a review by Borg Xuereb et al,^25^ time has been reported as a key challenge as well. No need for regular coagulation monitoring was a major reason for physicians to prefer DOACs over VKAs.^31^ The costs of DOACs compared to VKAs were was not the decisive factor but taking into consideration when deciding against DOACs as in other countries as well.^29,31^ Participants wished for a broader calculation on costs of different OACs. This broader calculation should include, for example, the costs associated with INR monitoring. Otherwise, it would merely result in a shift of costs from the medication budget to the workload of practicing physicians. Similar ideas were also mentioned by physicians in a study of Murphy et al^32^ A German study including these costs still showed a cost advantage of VKA versus DOACs, however INR monitoring was done here by patient self-testing.^33^

The preferences of patients have a large impact on prescribing behaviour. In Germany, patients can freely choose their physicians. If they are dissatisfied with the treatment, they may seek out another physician. Fulfilling the patients’ wishes was thus given higher priority by the participants than potential sanctions. The influence of patient preferences was also demonstrated in another sub-study of the *WirtMed study*, which investigated how new medications spread in the market.^34^ Furthermore, patient characteristics influence the choice of DOACs versus VKAs. As opposed to other studies,^28^ in our study it was not primarily the pharmacological distinctions, but rather considerations of adherence and the need for regular INR monitoring that factored in. DOACs offer the advantage of not requiring monitoring and therefore regular visits to the GPs’ offices are obsolete. At the same time, not being able to control the degree of anticoagulation is the biggest disadvantage, causing uncertainty and reluctance on the physicians’ side.^35^ Reports indicate that generally, approximately 50% of patients do not adhere to their prescribed medication regimen.^36,37^ However, taking DOACs as prescribed is crucial for them to be effective. In Germany, apixaban is the DOAC most often prescribed which needs to be taken twice daily.^38^ Andrade et al^39^ showed that patients were more likely to adhere to once-daily compared to twice-daily OACs. Overall, it remains uncertain whether medication adherence is superior with DOACs compared to VKAs.^40-42^ Moreover, in Germany a large proportion of patients with atrial fibrillation are treated with low-dose DOACs.^6^ Some participants raised concerns about the effectiveness and safety of DOACs in the older patient population, given their exclusion from RCTs, a concern confirmed by studies.^43^ A real-world analysis showed that the VKA phenprocoumon was associated with fewer thromboembolic events and a lower risk of death than low-dose DOACs.^5^ Internationally, RCT mostly focus on Warfarin and DOACs. For the in Germany mostly prescribed VKA phenprocoumon, only real world data exists. The real-world studies mostly showed near equivalence of efficacy of Phenprocoumon and DOACs.^5,6^

Switching patients from VKAs to DOACs and the higher initiation rate of DOACs will consequently lead to an increased number of patients with DOACs in the future. In a German survey the estimated ratio of prescriptions for DOACs and VKA was 2.9 (=68.6%/23.5%),^28^ meaning that most physicians would not meet the target set by the WSV. As in other countries the VKA Warfarin is mostly prescribed, no direct comparison to other countries can be drawn. However, it can be seen that for the treatment of cardiovascular diseases in particular, there are considerably more prescriptions in Germany than in other European countries.^44^

The targets are continuously changing, as generics, for example, enter the market, or new studies and updated guidelines are published in the meantime. At the beginning of 2020, the WSV itself was revised (WSV 3.0). The target quotas for DOACs were lowered due to changes in the reality of market and supply. Some interviewed GPs commented on being less satisfied with the revision.^17^ Starting 01.04.2024, there was a temporary suspension of anticoagulant prescription targets as the date of generics market entry was not exactly known.^45^

### Strengths and Limitations

This study subproject of the *WirtMed study* aimed to analyze the perception of GPs that have an overall prescribing target achievement of ⩾90% over several quarters. Physicians who prescribed cost-inefficiently over several quarters were not interviewed here and are part of another subproject of the *WirtMed study*. However, a target achievement of less than 100% can already lead to sanctions for physicians. This does not mean that we have only included physicians who perform exceptionally well in our study, which ensures that our sample is not solely composed of high performers. Also, concerning DOACs, the participating GPs all failed to achieve the target. The results may therefore also apply to other physicians who reach a total target achievement below 90%.

Our sample size was quite low. Overall, a sample of 65 physicians with different specialities was planned for the WirtMed-study. As it was not initially planned to focus on the prescription of the DOACs from the GPs’ perspective, no sample size was planned specifically for this question. We could focus on a topic that emerged inductively from the data which is a strength of the qualitative design. On the other hand, relevant aspects may not have been addressed and considered during the interviews and (small) group discussions, as DOACs and VKAs were not the main objects of the study. The findings of this study are underpinned by the TDF. Using this framework helped to assess the possible influences on behaviour methodologically. As the analysis was a secondary analysis the TDF was not used during the development of the guides. Hence, the questions were not based on the domains. However, our open analysis allowed the relevant domains to be identified.

## Conclusion

Having to achieve a target of prescribing VKAs as recommended drugs over DOACs, participating GPs mentioned to be caught between economic requirements, patients’ wishes, and pressure from secondary care / hospitals. Generally, they accept the necessity of cost control. However, they purportedly rate other factors like time constraints, patient satisfaction, and therapy adherence as overriding factors when it comes to prescription decision. The WSV implicitly assumes that GP prescribers can make independent decisions regarding the choice between DOACs and VKAs. However, it appears that their freedom may be constrained by other players. As OAC seems to be mostly indicated in the hospital, and as physicians almost exclusively prescribe DOACs there, participating GPs feel widely overchallenged in keeping the targets set by the WSV.

## Supplemental Material

sj-docx-1-his-10.1177_11786329251341083 – Supplemental material for Prescribing Vitamin-K-Antagonists Versus Direct Oral Anticoagulants Among Bavarian General Practitioners: A Qualitative Study Supplemental material, sj-docx-1-his-10.1177_11786329251341083 for Prescribing Vitamin-K-Antagonists Versus Direct Oral Anticoagulants Among Bavarian General Practitioners: A Qualitative Study by Nikoletta Zeschick, Julia Gollnick, Julia Muth, Franziska Hörbrand, Peter Killian, Norbert Donner-Banzhoff, Thomas Kühlein and Maria Sebastião in Health Services Insights
