# Supplementary material for: Prescribing Vitamin-K-Antagonists Versus Direct Oral Anticoagulants Among Bavarian General Practitioners: A Qualitative Study
Source: Health Serv Insights. 2025 Jun 21;18:11786329251341083. doi: 10.1177/11786329251341083 (PMC12182603; doi:10.1177/11786329251341083)
Supplement: sj-docx-1-his-10.1177_11786329251341083 – Supplemental material for Prescribing Vitamin-K-Antagonists Versus Direct Oral Anticoagulants Among Bavarian General Practitioners: A Qualitative Study [file sj-docx-1-his-10.1177_11786329251341083.docx]

**Additional file 1**

*Interview and Focus Group Guide*

# General information on the person and practical experience

| First, we would like you to introduce yourself. (Focus group: This helps us to get to know you all, of course, and at the same time it is important for the transcription later to get a voice recording of each of you at the beginning.) |
| --- |
| **Introduce yourself in a few words and tell us where your practice is and what typical patient cases look like for you.** |
| - For example age, gender, clinical conditions |

# General information on prescription behavior

Now it is about therapy options and drug prescriptions.

*Example: In general, when a patient comes to your practice, you make a diagnosis and he is supposed to be given a drug:*

**What criteria are important for you when choosing between different drug therapies?**

*Provide examples after the physician has explained his initial answers:*

- Mechanism of action

- Effect

- Prescription of the guidelines

- Manufacturer

- Prices

- Personal experience

- Pharmaceutical representatives

- Further education

- Congresses

- Patient wishes

- Former recourse claims

- Concomitant medications

- Comorbidity

- Studies

**To what extent do patients and their wishes influence your drug prescription?**

*- What kind of wishes are these, for example?*

*- How do you deal with them in everyday life? (Keyword: Aut idem)*

*- How do your patients react when you do not prescribe the preparation the patient wants? (Keyword: Adherence problems)*

*- How often does it happen?*

**When specific questions arise about a medication, how do you proceed?***

Here, above all: efficacy, safety, cost-effectiveness - how do you inform yourself?

***New medications are regularly approved on the market, and you have to decide whether or not to give them to your patients. How do you proceed?***

**And how do you deal with therapies initiated by the hospital / other specialists??**

- *What effects do therapies initiated by other specialists have on your prescribing behavior?*
- *Are they continued?*

# **General situation and** “Wirkstoffvereinbarung” (WSV; English: Active Substance Agreement)

**There was an article from the Association of Statutory Health Insurance Physicians (ASHIP) Hamburg that summarizes the active substance agreement as follows:**

In the former system, the so-called “Richtgrößenprüfung” (*English*: “Prescribing Target Auditing” scheme), physicians had responsibility for drug costs - a fundamental design flaw.

The new model is no longer about absolute costs but about DDD quotas and shares of generics and lead substances.

With a twist: The physician is only responsible for what he can influence himself - he chooses the right active substance.

"The model shifts the actual cost question away from the physicians and to where it belongs, namely to the health insurance funds".

**To what extent does this fit with your experience with the WSV? How do you assess this statement?**

*- To what extent has your prescribing behavior changed since then?*

*- What effects do you expect the Bavarian Agreement on Active Substances to have on your freedom of therapy/therapeutic sovereignty?*

*- Advantages/disadvantages compared to earlier procedures?*

*- What adjectives can you use to describe them? (Positive as well as negative descriptions; restrictive, helpful, supportive, safeguarding).*

*- Are there any diseases/indications that you would treat differently than the active substance agreement prescribes?*

*- How do you manage to keep to your goals within the framework of the Bavarian Agreement on Active Substances?*

- - Have you ever taken advantage of a counseling offer for prescription assistance?
  - If yes, how did this take place?

**How satisfied are you currently with your drug prescribing situation?**

- *How do you rate the satisfaction of your colleagues/other specialists with their prescribing situation?*

**What do you expect from the ASHIP in Bavaria? Do they meet these expectations?**

**What would make prescribing drugs easier for you? How could the system of the WSV be improved?**

After all, the WSV was introduced for a reason and is based on the efficiency principle determined by law. **Where do you think this development will lead to in the next five to ten years?**

**Presentation of the paragraphs:**

§ 12 SGB V Cost-effectiveness requirement
The service must be sufficient, appropriate and economical; it must not exceed what is necessary. Insured persons may not claim benefits that are not necessary or uneconomical, the service providers may not provide them and the health insurance funds may not approve them.

§ 106 SGB V Performance Audit

The health insurance funds and the associations of SHI-accredited physicians shall monitor the efficiency of SHI-accredited medical care through consultations and audits.

**In medical care, there is a lot of talk about costs and economic efficiency, which is also to be audited according to the law. How do you feel about this personally?***

- *Work motivation*
- *Emotion*

**In your eyes, are the terms "economic efficiency" and "quality" compatible in the field of drug therapy?**

# Conclusion & bottom line

Finally, we would like to leave room for other aspects that might still be important to you.

**What was your motivation to participate?***

**Are there any other aspects that you would like to address?**

After we have looked at these different facets, we would like to draw a resume.

**When you think about your daily work now, what aspects do you see critically / what would you like to change if you could?***

**And what do you particularly enjoy?***

**To sum up the past hour, how do you rate the WSV?**

**Conclusion: Thank you very much for your participation!**
